# The active effect of *Rhizophagus irregularis* inoculants on maize endophytic bacteria community

**DOI:** 10.1002/imo2.23

**Published:** 2024-08-17

**Authors:** Jin Chen, Keqing Lin, Tao Huang, Xiaowan Geng, Zishan Li, Boyan Wang, Qingchen Xiao, Xiaoyu Li

**Affiliations:** ^1^ School of Life Sciences Anhui Agricultural University Hefei China; ^2^ National Engineering Laboratory of Crop Stress Resistance Breeding Anhui Agricultural University Hefei China; ^3^ Key Laboratory of Crop Stress Resistance and High‐Quality Biology of Anhui Province Anhui Agricultural University Hefei China

## Abstract

Little has been reported on the effect of arbuscular mycorrhizal fungi (AMF) inoculants from in vitro dual culture system on the growth of maize and its endophytic microbial community. Our results suggest that AMF inoculants play an important role in influencing maize growth and diversity of endophytic bacterial communities. AMF inoculants significantly promote maize growth, especially AMF inoculants with modified Strullu‐Romand (MSR) medium. These inoculants also significantly increased the diversity of endophytic microbial communities, especially the abundance of beneficial bacterial flora, thus positively affecting maize growth. This study reveals the utility of AMF inoculants from in vitro dual culture system, which provides a basis for the development of environmentally friendly inoculants.

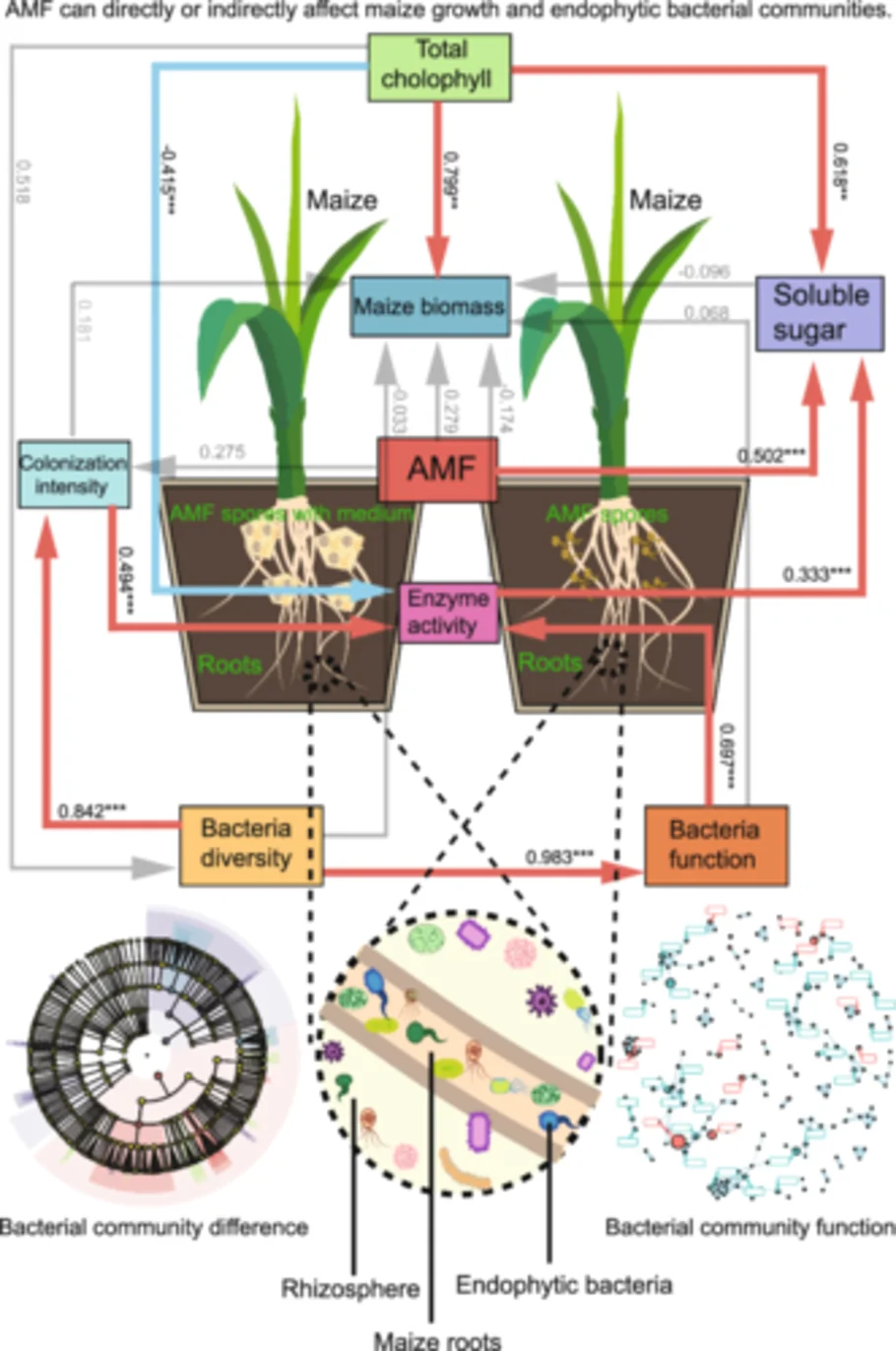

Arbuscular mycorrhizal fungi (AMF) are vital in agricultural and natural ecosystems, forming a symbiotic relationship with over 80% terrestrial plants. They have been suggested as an ecological additive to enhance plant growth in both normal and stress conditions [1]. For example, AMF enhances plant resistance to pathogens, alters plant morphology, physiology, biochemistry, and gene expression to improve the rhizosphere microenvironment, which in turn impacts plant‐feeding insects [2]. AMF propagules typically consist of spores, hyphae, and infected plant root segments. Currently, the majority (about 68%) are using the mixture of these propagules as a microbial inoculant in scientific research and agricultural production to improve crop yields, to help host plants resist stress or to improve the soil environment, while only 14% utilize AMF spores [3]. The prevalent culture systems for AMF are pot culture and in vitro dual culture, where germinated spores and host materials are inoculated on a sterile modified Strullu‐Romand (MSR) medium to enable AMF mycelium to invade the roots, acquire nutrients, and complete the life cycle [4]. This in vitro dual culture system improves the efficiency of AMF spore production and avoids the risk of soil contamination compared to traditional pot culture [5], and it is also small and light when air‐dried, containing about 6000 spores per gram, making it easy to store at room temperature for long period. However, there are many studies investigating the effects of pot‐expanded AMF inoculum on crops, but few studies investigating the effects of inoculation with AMF from in vitro dual culture systems.

Maize (*Zea mays* L.), a globally important food and feed crop, faces multiple stresses caused by industrialization, heavy metal pollution, and soil salinization. Endophytic bacteria, as an important microbial community in plants, play a key role in plant growth regulation and stress responses [6]. These bacteria promote plant growth by indirectly protecting against soil‐borne diseases and directly aiding in nutrient absorption. Although the roles of AMF and endophytic bacteria in plant growth have been extensively studied, little has been reported on the effects of AMF inoculum from in vitro dual culture systems on maize growth and its endophytic microbial community. To fill this knowledge gap, we used high‐throughput sequencing technology to quantify the effects of AMF co‐inoculation and in vitro dual culture on the endophytic bacterial community under different air‐drying conditions (Figure 1A) and thus assessed their growth‐promoting effects on maize. We found that the inoculation of AMF artificially air‐dried with medium (ADM) treatment had the best growth‐promoting effect on plant height and dry weight, followed by the inoculation of AMF naturally ADM (NDM) treatment (see Table S1 for details of treatment design). Although the group of artificially air‐dried treatment recruited more endophytic bacteria with increased number of beneficial endophytes.

**Figure 1:**
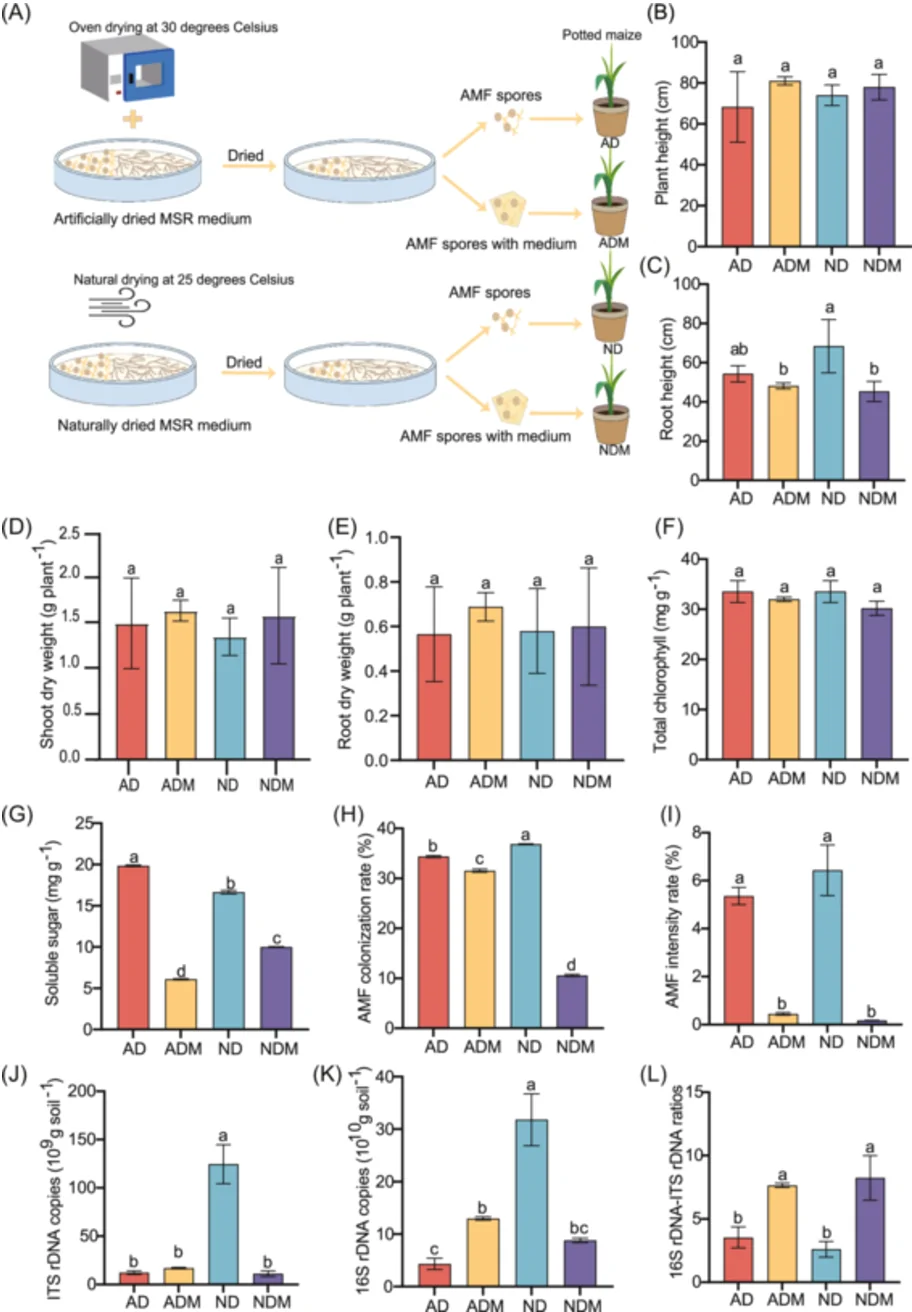
Analysis of maize growth under different arbuscular mycorrhizal fungi (AMF) treatments. (A) The flow chart of different steps of spore acquisition and maize planting. (B, C) The length of maize shoot and primary root. (D, E) Dry weight of maize shoot and root. (F, G) Total chlorophyll levels and soluble sugar content in each treatment at 70 days after maize planting. (H, I) Mycorrhizal colonization rate and colonization intensity for each treatment. (J–L) The root internal transcribed spacers (ITS) ribosomal DNA (rDNA) copies, 16S rDNA copies, 16S rDNA‐ITS rDNA ratios under different treatments. Three replicates for each treatment, all data were presented as mean ± SEM (*n* ≥ 3 biological replicates). Different letters indicate significant differences (*p* < 0.05). AD, artificially dried; ADM, artificially dried with medium; ND, naturally dried; NDM, naturally dried with medium.

## 1 RESULTS AND DISCUSSION

The symbiotic relationship between AMF and plants not only promotes plant nutrient uptake but also enhances plant adaptation in the face of various stresses [7]. By assessing maize traits after inoculation with *Rhizophagus irregularis* (Ri) spores and MSR medium from two air‐drying methods, we showed that there were significant differences in maize traits between these treatments (Figure S1). Compared with the uninoculated control, inoculation with AMF significantly improved maize growth (*p* < 0.05), including shoot and root height, chlorophyll content, and fresh and dry weights (Figure 1B–F and Figure S2 and Table S2), with the ADM treatment having a more pronounced growth‐promoting effect on plant height and dry weight. In addition, inoculation with AMF spores alone increased the colonization rate and intensity of AMF, as well as the soluble sugar content of maize (Figure 1G–I).

Several studies have shown that phosphatase activity in plants was significantly increased after AMF inoculation [8]. Meanwhile, the activities of key soil enzymes, such as S‐NAG (soil *N*‐acetyl‐β‐d‐glucosidase) and phosphatase, were also significantly elevated in the rhizosphere soil inoculated with AMF [9]. These soil enzymes are important indicators of soil health, reflecting the extent and direction of biochemical processes [10]. Notably, when the MSR medium was co‐inoculated with AMF, substrate pH and the activities of three enzymes (soil neutral phosphatase, soil *n*‐acetyl‐d‐glucosaminidase, and soil β‐glucosyl enzyme) were significantly reduced compared to that co‐inoculated with AMF spores alone (Figure S3). This aligns with the observations in Figure 1H,I, that co‐inoculation reduced AMF colonization rates and intensities. The infection intensity reflects the weighted average of infection severity across all infected root segments and a higher infection intensity indicates better plant reproduction after AMF infection. In the ADM treatment, a 31.55% infection rate indicates successful AMF colonization. However, infection intensities in the ADM and NDM treatments were significantly lower than those in the inoculation of AMF artificially air‐dried (AD) and inoculation of AMF naturally air‐dried (ND) groups (Figure 1H,I). This difference may be attributed to the encapsulation of the medium, which hinders the contact between AMF spores and the plant root system, thus affecting AMF reproduction. These results suggest that the adding of MSR medium hinders AMF colonization on host plants.

Additionally, we observed that inoculation with AMF significantly increased the abundance of endophytic bacteria and fungi in maize roots (Figure 1J–L and Table S3), as well as the endophytic microbial community in maize roots, especially the bacterial community, detected by 16S ribosomal DNA (rDNA)‐internal transcribed spacers rDNA ratio analysis (Figure 1L). Therefore, we further investigated the influence of AMF inoculation on the endophytic bacterial community in maize.

**Figure 2:**
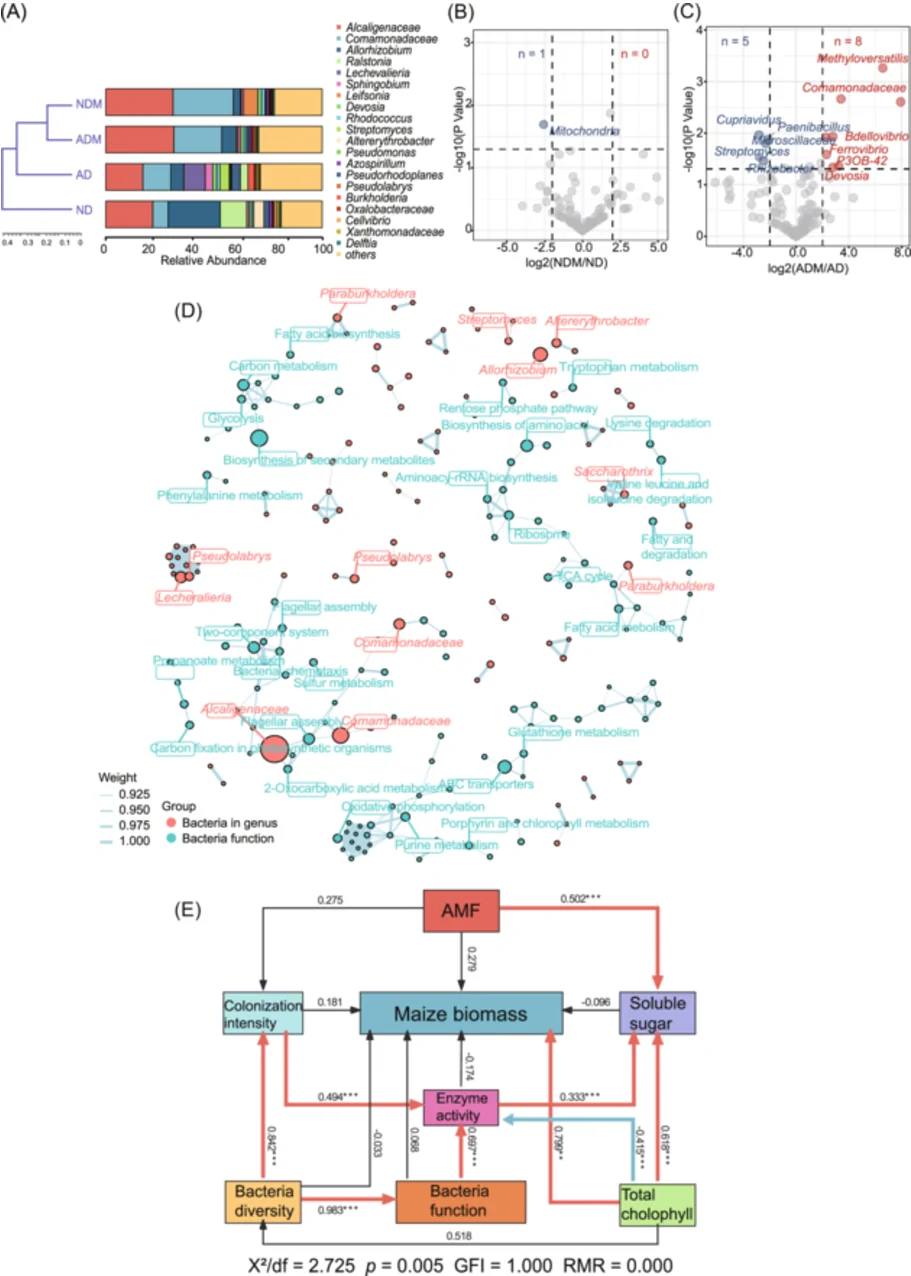
Composition and differences of maize endophytes in different treatments and potential key factors affecting plant biomass. (A) The cluster analysis of the bacteria community by weighted distances. (B, C) Bacterial communities between different treatments significantly increased or decreased at fixed thresholds (fold change > 4 and −log *p* > 0.05) in the volcano plots. (D) Correlation network analysis reveals the relationship between bacterial function and genus‐level microbial taxa. Connections represent strong (*r* > 0.8) and significant (*p* < 0.01) correlations. The size of each node is proportional to the number of connections. (E) The structural equation model (SEM) describes the relationship between different factors. Red arrows represent significant positive correlations, blue arrows represent significant negative correlations, and black arrows represent non‐significant.

It is worth noting that endophytic microbial communities and crop characteristics can be influenced by various factors such as soil chemical properties and agricultural practices [10, 11], including soil moisture, organic matter, and pH [12]. Similarly, the redundancy analysis in Figure S4 indicated that the inoculation of AMF was the primary factor influencing bacterial differences, followed by pH and S‐NAG. Inoculation with AMF notably enhanced the abundance of endophytic bacteria in maize roots (Table S3). This aligns with previous studies showing AMF inoculation alters rhizosphere bacterial composition, boosting beneficial bacteria abundance [13]. Specifically, AMF inoculation promoted an increase of bacteria populations with specific functions, such as *Dyella*, *Ruminococcus*, *Allorhizobium*, and *Pseudolabrys*, which play important roles in hydrolase synthesis, heavy metal transport, and metabolite transport [14]. Therefore, inoculation with AMF is expected to be an effective way to establish and enrich endophytic microbial communities [15]. Notably, the number of endophytic bacterial genera was higher in the artificially dried treatments, especially in the ADM group, which contained a total of 389 bacterial genera (Figure S5). Further analyses showed that the addition of AMF to the MSR medium significantly increased the abundance of *Alcaligenaceae* and *Comamonadaceae* in the substrate (Figure 2A). *Comamonadacea* promotes the growth of maize at low temperatures [16], while *Alcaligenaceae*, as a microbial fertilizer, degrades harmful soil substances and produces indoleacetic acid [17]. Therefore, ADM treatment and NDM treatment were more favorable for the recruitment of beneficial bacterial communities.

Using linear discriminant analysis effect size analyses, we further identified significant differences in core microbial communities between different treatments, with a total of 39 distinct species displaying linear discriminant analysis values exceeding 3.5 (Figure S6A). Cladograms highlighted notable variations in biomarkers among different treatments, with *Proteobacteria* prevailing in all treatments, while AD and ADM exhibited a higher abundance of *Actinobacteriota* and *Myxococcota* (Figure S6B). To gain further insights into the shifts in bacterial communities across treatments, volcano analysis was carried out with groups subjected to different air‐drying methods (Figure 2B,C). Notably, *Mitochondria* were less abundant in NDM compared to ND (Figure 2B and Figure S7A). However, in the ADM, the observed main bacteria were *Comamonadaceae* and *Methyloversatilis*, in contrast with the more abundant *Cupriavidus* in the AD (Figure 2C and Figure S7B). Furthermore, the function analysis and correlation network analysis revealed a significant association between bacteria *Alcaligenacaea* and *Comamandaceae* with biological functions including Flagellar assembly, 2‐Oxocarboxylic acid metabolism, sulfur metabolism, carbon fixation in photosynthetic organisms, and two‐component system (Figure 2D and Figure S8). In particular, 2‐Oxocarboxylic acid and sulfur metabolism are crucial for maintaining cellular structure and function, energy metabolism, and cell signaling [18]. Photosynthetic carbon fixation is a vital mechanism for plants to enhance yield [19]. The addition of AMF inoculant with MSR medium could significantly increase the abundance of *Alcaligenacaea* and *Comamondaceae* in maize roots, enhancing 2‐Oxocarboxylic acid, sulfur metabolism, and photosynthetic carbon fixation, thus stimulating energy metabolism and photosynthesis [20]. This was also verified by Mantel analysis, where AMF (spore density of AMF in different treatments) was positively correlated with bacterial function, and maize biomass (aboveground and belowground dry weight) was positively correlated with total chlorophyll and colonization rate (Figure S9). Further analysis using structural equation model (SEM) revealed that AMF directly impacted soluble sugar content, while total chlorophyll content of maize directly influenced both maize biomass and soluble sugar content. Interestingly, the content of soluble sugars had a negative correlation with maize biomass, consistent with the findings in Figure 1G where the soluble sugar content of NDM and ADM was significantly lower than that of AD and ND. This might explain why there were minimal differences in fresh weight, plant height, and chlorophyll content among these treatments, despite the significantly lower AMF colonization rate in NDM and ADM compared to AD and ND. This may be due to a positive correlation between colonization intensity and soluble sugar content (Figure 2E), where higher colonization intensity may have increased soluble sugar content, but excessive soluble sugar content inhibited maize growth, while chlorophyll played a compensatory role in sustaining maize growth. In addition, changes of fungal communities may also contribute to maize growth by adding AMF inoculants, which needs to be explored further.

## 2 CONCLUSIONS

In conclusion, our study found that single inoculation of AMF spore treatments had more favorable spore activity, but treatments co‐inoculated with MSR medium were more effective in promoting maize plant height and dry weight. And such inoculants co‐inoculated with MSR medium could positively affect maize growth by significantly increasing the abundance of beneficial endophytic bacteria populations, such as *Alcaligenaceae* and *Comamonadaceae*. This provides a basis for the development of an environmentally friendly inoculant, but the specific response of the fungal community and the effectiveness of this inoculant for practical application in field environments still need to be further explored and verified.

## 3 METHODS

The comprehensive methodology, encompassing experimental procedures, sequencing protocol, data processing techniques for sequencing data, and approaches for data analysis, is available in the Supporting Information.

## AUTHOR CONTRIBUTIONS

Jin Chen, Xiaoyu Li, and Tao Huang conceived and designed the study. Tao Huang, Keqing Lin, Boyan Wang, and Qingchen Xiao collected the samples and performed the experiments. Keqing Lin, Xiaowan Geng, and Zishan Li analyzed the data, and wrote the manuscript. Jin Chen and Xiaoyu Li revised the manuscript. All authors have read the final manuscript and approved it for publication.

## CONFLICT OF INTEREST STATEMENT

The authors declare no conflict of interest.

## ETHICS STATEMENT

No animals or humans were involved in this study.

## Supporting information


**Figure S1:** Growth of maize after 70 days in different treatments.
**Figure S2:** Fresh weight of maize in different treatments.
**Figure S3:** Soil chemical properties in different treatments.
**Figure S4:** Redundancy analysis (RDA) illuminating soil chemical properties and bacteria community composition at the phylum level.
**Figure S5:** Venn diagram of endophytic bacterial ASV in each treatment.
**Figure S6:** Linear discriminant analysis effect size (LEfSe) analysis revealed significant differences endophytic bacteria in different groups.
**Figure S7:** The relative abundance of bacterial genera with differences is shown in the bubble diagram.
**Figure S8:** Functional composition of endophytic bacteria in roots of maize in different treatments.
**Figure S9:** Mantel test revealed the correlation between bacterial diversity and function, soil physical and chemical properties, maize physiological indexes, and maize biomass and AMF.

**Table S1:** Physiological indices of maize in different treatments.
**Table S2:** AMF colonization and microbial copy number in maize in different treatments.
**Table S3:** Different inoculation treatments of maize roots.

## Data Availability

All the sequencing data have been deposited in GSA under submission number SUBCRA027928, BioProject accession number CRA017430 (https://bigd.big.ac.cn/gsa/browse/CRA017430). The data and scripts used are saved in GitHub, https://github.com/lkq0616/Rhizophagus-irregularis-impact-on-maize-endobacteria-community. Supporting Information (methods, figures, tables, graphical abstract, slides, videos, Chinese translated version, and update materials) may be found in the online DOI or iMetaOmics, http://www.imeta.science/imetaomics/.
