# Supplementary material for: The active effect of Rhizophagus irregularis inoculants on maize endophytic bacteria community
Source: IMetaOmics. 2024 Aug 17;1(1):e23. doi: 10.1002/imo2.23 (PMC12806334; doi:10.1002/imo2.23)
Supplement: Supplementary file 1 — Figure S1: Growth of maize after 70 days in different treatments. Figure S2: Fresh weight of maize in different treatments. Figure S3: Soil chemical properties in different treatments. Figure S4: Redundancy analysis (RDA) illuminating soil chemical properties and bacteria community composition at the phylum level. Figure S5: Venn diagram of endophytic bacterial ASV in each treatment. Figure S6: Linear discriminant analysis effect size (LEfSe) analysis revealed significant differences endophytic bacteria in different groups. Figure S7: The relative abundance of bacterial genera with differences is shown in the bubble diagram. Figure S8: Functional composition of endophytic bacteria in roots of maize in different treatments. Figure S9: Mantel test revealed the correlation between bacterial diversity and function, soil physical and chemical properties, maize physiological indexes, and maize biomass and AMF. [file IMO2-1-e23-s001.docx]

**Supporting information to：**

**The active effect of *Rhizophagus irregularis* inoculants on maize endophytic bacteria** **community**

**Running title:** *Rhizophagus irregularis* impact on maize endobacteria community

Jin Chen^1,2,3#^, Keqing Lin^1,2,3#^, Tao Huang^1,2,3^, Xiaowan Geng^1,2,3^, Zishan Li^1,2,3^, Boyan Wang^1,2,3^, Qingchen Xiao^1,2,3^, Xiaoyu Li^1,2,3*^

^1^ School of Life Sciences, Anhui Agricultural University, Hefei, 230036, China.

^2^ National Engineering Laboratory of Crop Stress Resistance Breeding, Anhui Agricultural University, Hefei, 230036, China.

^3^ Key Laboratory of Crop Stress Resistance and High-Quality Biology of Anhui Province, Anhui Agricultural University, Hefei 230036, China.

^#^These authors contributed equally: Jin Chen, Keqing Lin

*Correspondence: [lixiaoyu@ahau.edu.cn](mailto:lixiaoyu@ahau.edu.cn) (Xiaoyu Li)

**Supplemental Text:**

**MATERIALS AND METHODS**

**Preparation of Material**

The growth medium used in the experiment was prepared from purchased river sand and zeolite. After washing and drying, the river sand and zeolite were mixed in the ratio (2:1 v/v), and sterilized at 121 °C for 40 min. The growth medium was a weakly alkaline substrate with a pH of 7.15.

The plant material used in this study was maize (AN591) from Anhui Agricultural University. The variety has moderate maturity and good summer sowing performance, which is suitable for large-scale promotion and application. Maize seeds were disinfected with 12 % (v/v) Huawang disinfectant (Contains 10 % NaClO solution) for 10 min. The sterile seeds were placed on a wet germination paper at an interval of 1 cm, and placed in a light incubator (16 h light, 8 h dark, 25 °C) for 5-7 d. The seedlings of the same size were selected and transplanted into pots.

The AMF strain selected in this experiment was *Rhizophagus irregularis* (*R. irregularis*) DOAM197198 from Anhui Agricultural University. The large-scale production of *R.irregularis* in our laboratory is mainly through *in vitro* dual culture. The *in vitro* dual culture system of fungi and carrot-induced epidermal hair roots was carried out on a modified Strullu-Romand (MSR) medium plate. The plates were incubated in the dark at 25 °C for 4 months.

**Experimental design and procedure**

This experiment was conducted in a glass greenhouse at Anhui Agricultural University, Anhui Province, China (31°52'N, 117°14'E). The climate of the study area is suitable for the growth of the plants. In the *in vitro* dual culture system, the incubation temperature for AMF is typically set at 25°C. However, this temperature often prolongs the air-drying process of the medium. To expedite the air-drying and ensure optimal spore activity, we experimented with placing the medium inoculated with AMF in an oven set at 30°C for rapid drying. Therefore, this experiment was designed with a total of four different treatments: Inoculation of AMF spores without medium selected from artificially dried MSR medium (AD), inoculation of AMF spores with medium selected from artificially dried MSR medium (ADM), inoculation of AMF spores without medium selected from naturally dried MSR medium (ND), inoculation of AMF spores with medium selected from naturally dried MSR medium (NDM), see Table S1 for details. Individual AMF spores were collected under a microscope by crushing air-dried MSR medium with water in a juicer and filtering through a 70-mesh sieve. AMF spores carrying MSR medium were crushed with a juicer and collected under a microscope. All maize was planted in pots (Diameter: 23.3 cm, Depth: 25 cm), and each pot treatment was set in triplicate, each pot has one maize plant. There was 4 kg (oven-dried weight basis) of substrate in each pot, equivalent to about a 20 cm-deep substrate layer. The spores of *R.irregularis* were isolated from *in vitro* dual culture and counted under a binocular microscope to ensure that the number of spores inoculated in the four treatments of AD, ADM, ND and NDM was about 120 per maize plant. All treatments were placed under the same greenhouse conditions. During the experiment, low - P Hoagland nutrient solution (the concentration of P was 0.1 mM) or deionized water was added to the weight of the growth substrate every week, so that the weight moisture content of the growth substrate was maintained at 18 % -20 % (water holding capacity was 70 %).

**Sample collection and analysis**

In this study, maize plants were harvested after 70 days of growth. The roots and leaves of each maize plant were harvested separately, and the surface growth medium was rinsed with a running faucet. All maize plants were selected as samples for measuring physiological parameters. The length of aboveground and belowground parts of plants was measured with a ruler, while their fresh biomass was measured with an electronic balance, and dry biomass was measured with an electronic balance after drying. After measuring the basic physiological traits of plants, samples were collected, sealed with plastic bags, and transported to the laboratory ice box for storage within 24 hours. The leaves were placed at 4 °C for the analysis of physical and chemical indicators. The chlorophyll content was determined by ethanol extraction method [1]. The soluble sugar content of fresh maize leaves was determined by Beijing Soleibao kit and anthrone method. 0.1 g of fresh maize leaves in the same position was weighed to determine the soluble sugar content of fresh maize leaves [2].

After surface moisture drying, the roots were divided into two parts. A part of the surface was sterilized according to the method of Coombs et al., and stored at -80 °C [3]. The other part of the root was divided into about 1 cm, washed in 10 % KOH solution, and stained with trypan blue staining. The infection rate and intensity of AMF were determined according to the evaluation method of Trouvelot et al [4], infection rate (F%) and intensity of infection (M%) were calculated according to the following formulae:

*F% = (Number of AMF-infected root segments in the sample / Total number of root segments in the sample)*100%*

*M% = (95*n5+70*n4+30*n3+5*n2+n1 / Total number of root segments in the sample)*100%*

n5, n4, n3, n2, n1 represent the number of root segments with infestation intensity greater than 90%, 70%, 50%, 10% and 1% in each treatment group.

The growing media in each pot was placed in separate sealed bags, marked and immediately shipped back to the laboratory. Each growing media sample was divided into two subsamples. One subsample was stored at 4 ℃ for microscopic examination of AM spore density, while another was air dried to constant weight for physicochemical properties determination. The pH of air-dried samples of growing media was measured by a pH meter (PB-10, Sartorius, Germany) in a 1: 2.5 substrate-to-water ratio. The activities of β-1,4-glucosidase (BG), N-acetyl-β-D-glucosaminidase (NAG) and neutral phosphodiesterase (NP) involved in C, N, and P cycles were analyzed according to Tabatabai [5]. The samples were spiked in a 96-well microplate reader according to the manufacturer's instructions, and the absorbance at 400, 400, and 660 nm for each enzyme was measured.

**DNA extraction and Illumina MiSeq**

We used the E. Z. N. A™ Mag‐Bind Substrate DNA Kit (Bio-Teke Corporation, Beijing, China) to extract DNA of root endophytic bacteria and fungi from each sample. DNA quality and concentration were measured using Agarose gel electrophoresis (1 %) and a Qubit 3.0 Fluorometer (Life Technologies). Total 16S and internal transcribed spacers (ITS) rDNA copy numbers of maize roots were determined using the ABI StepOne Plus® Real-Time PCR system (Applied Biosystems, Foster City, CA, USA) with universal bacterial primer pair 338F/518R and universal fungal primer pair ITS1F/ITS2R [6]. The 10 μL reaction mixtures contained 1μL template DNA, 5μL Maxima™ SYBR Green/ROX, 0.5μL forward primer, 0.5μL reverse primer and 3μL nuclease-free water.

The qualities and contents of the extracted DNA were verified using a NanoDrop ND-1000 ultra-violet visible (UV-Vis) spectrophotometer (NanoDrop Technologies, Wilmington, DE, USA), the sterilized root samples were sent for sequencing to Majorbio Biotechnology (Shanghai, China). Using Illumina MiSeq (PE300) sequencing platform, the composition of the endophytic bacterial community of maize roots were further explored by sequencing target amplicons. Bacterial V4-V5 hypervariable region of the 16S rRNA gene was amplified by PCR using the primers 515F (5′-GTGCCAGCMGCCGCGGTAA-3′), and using 528F(5-CGGGTAATTCCAGCTCCAA-3′) and 706R (3′-AATCCRAGAATTTCACCTCCAA-5′).

After sequencing was completed, paired-end sequences were spliced using FLASH (V1.2.7, <http://ccb.jhu.edu/software/FLASH>) [7]. Microbial Ecology (QIIME) v1.8.0 pipeline was used to separate each sample sequence from the raw reads based on the barcode, and the barcode sequence was trimmed to obtain the original data. After removing low-quality bases and linker contamination sequences, high-quality target sequences were filtered from the data for further analysis [8]. The raw sequencing reads were assigned to operational taxonomic units (ASVs). The Illumina MiSeq sequence data were submitted to the GSA (BioProject accession number CRA017430) database.

**Statistical analysis**

Analysis of variance (ANOVA) was performed on fresh weight, plant height, total chlorophyll, soluble sugar and mycorrhizal infection rate using SPSS Statistics 26 (SPSS Inc., Chicago, IL, USA). A posteriori comparison was made using Tukey tests (*p* < 0.05). The bar diagrams run on Graphpad Prism 9.0. Afterwards, the overlapping and enriched genera were visualized using Venn diagram (<https://www.bic.ac.cn/>). The bacterial abundance at the phylum level in each treatment group was demonstrated by hierarchical clustering and stacked histograms (<http://cloudtutu.com.cn/>). Linear discriminant analysis effect size (LEfSe) analysis (<https://www.majorbio.com/>) was used to detect the difference in relative abundance of bacteria, with an alpha value of 0.05 for factorial Kruskal-Wallis tests and an LDA score threshold of 3.5. Volcanic curve analysis ([https: //www.cloudtutu.com](http://cloudtutu.com.cn/)) was performed to detect enriched and depleted bacteria in different sample groups. PICRUSt2 was used to predict bacterial function and metabolic pathways using the KEGG database [9]. The network diagram was drawn using the Tutools platform (<http://cloudtutu.com.cn/>). The network analysis revealed the co-occurrence pattern of substrate bacterial function and substrate bacteria at the genus level. In addition, Mantel test showed the relationship between AMF, maize biomass and other physical and chemical factors. Advanced Cor link was performed using the OmicStudio tools (<https://www.omicstudio.cn/tool>). The relationships between AMF, soluble sugar, total chlorophyll, bacterial communities, and maize biomass were determined by structural equation modeling (SEM) using AMOS 23.0 [10]. The RDA analysis was drawn using the Tutools platform (<http://cloudtutu.com.cn/>).

**REFERENCE:**

1. Arnon Daniel I. 1949. “Copper enzymes in isolated chloroplasts. Polyphenoloxidase in Beta vulgaris.” *Plant Physiol* 24: 1. <https://doi.org/10.1104/pp.24.1.1>
2. Moretti, Ademar, Cintia L. Arias, Leandro A. Mozzoni, and Pengyin Chen, et al. 2020. “Workflow for the quantification of soluble and insoluble carbohydrates in soybean seed.” *Molecules* 25: 3806. <https://doi.org/10.3390/molecules25173806>
3. Coombs Justin T., and Christopher M. M. Franco. 2003. “Isolation and identification of actinobacteria from surface-sterilized wheat roots.” *Appl Environ Microb* 69: 5603-5608. <https://doi.org/10.1128/AEM.69.9.5603-5608.2003>
4. Trouvelot A, Kough J, and Gianinazzi-Pearson V. 1986. “Evaluation of VA infection levels in root systems. Research for estimation methods having a functional significance.” *Physiological and genetical aspects of Mycorrhizae* 217-221.
5. Tabatabai, M. A. 1994. “Soil Enzymes.” *Methods of Soil Analysis: Part 2-Microbiological and Biochemical Properties* 5: 775-833. <https://doi.org/10.2136/sssabookser5.2.c37>
6. Lane, D. J. 1991. “16S/23S rRNA sequencing. Nucleic acid techniques in bacterial systematics.” *Chichester: Wiley*: 125-175.
7. Liu, Yuansi, Lixue Gong, Xiaoying Mu, Ziqiu Zhang, Tiantian Zhou, and Songhe Zhang. 2020. “Characterization and co-occurrence of microbial community in epiphytic biofilms and surface sediments of wetlands with submersed macrophytes.” *Sci Total Environ* 715: 136950. <https://doi.org/10.1016/j.scitotenv.2020.136950>
8. Schloss, Patrick D. 2009. “A high-throughput DNA sequence aligner for microbial ecology studies.” *PloS one* 4: e8230. <https://doi.org/10.1371/journal.pone.0008230>
9. Langille, Morgan GI, Jesse Zaneveld, J. Gregory Caporaso, Daniel McDonald, Dan Knights, and Joshua A. Reyes, et al. 2013. “Predictive functional profiling of microbial communities using 16S rRNA marker gene sequences.” *Nat Biotechnol* 31: 814-821. <https://doi.org/10.1038/nbt.2676>
10. Chen, Jin, Zishan Li, Daolong Xu, Qingchen Xiao, Haijing Liu, and Xiaoyu Li, et al. 2023. “Patterns and drivers of microbiome in different rock surface soil under the volcanic extreme environment.” *iMeta* 2: e122. <https://doi.org/10.1002/imt2.122>

**
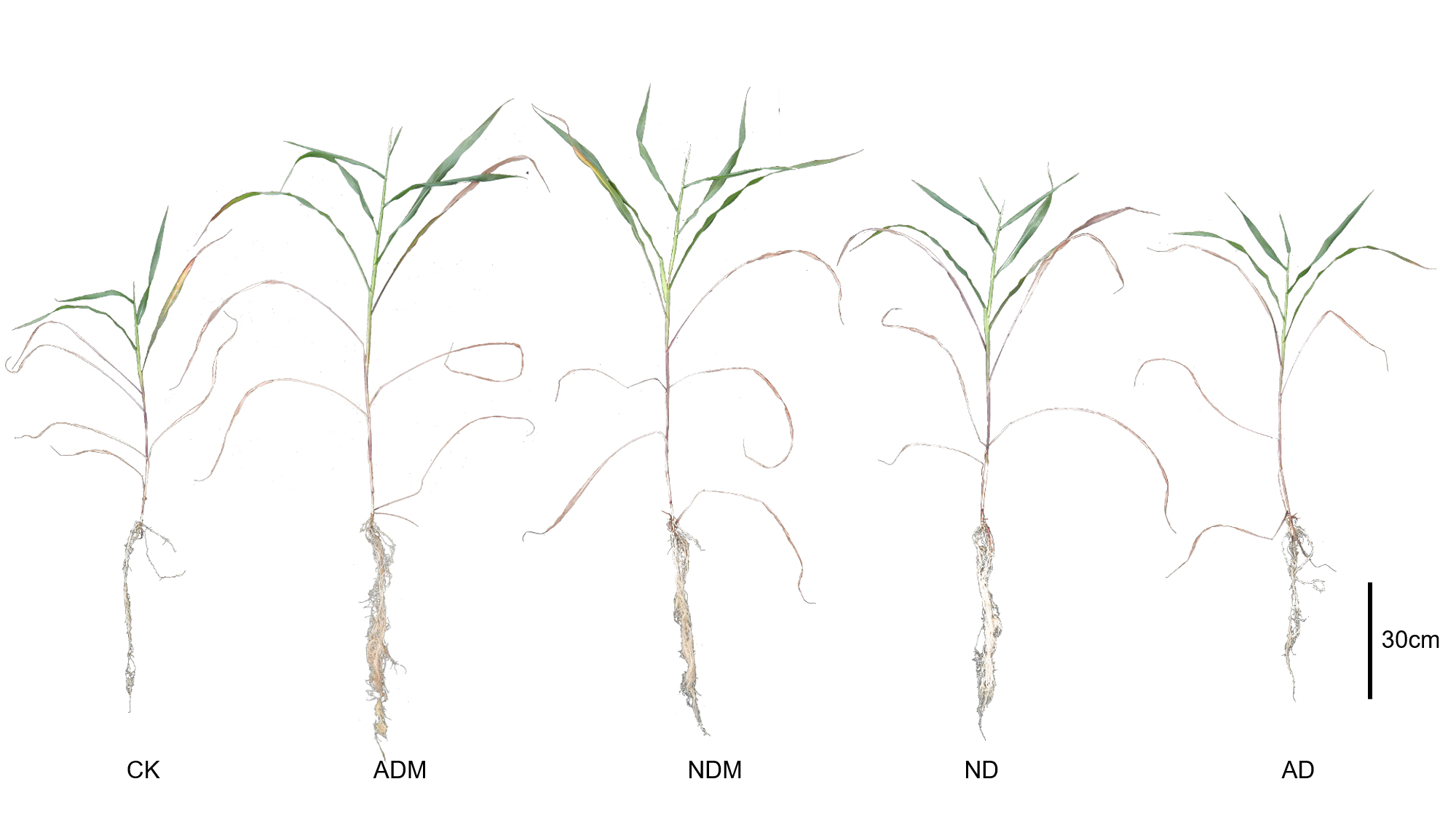
**

**Figure S1 Growth of maize after 70 days in different treatments.** CK, control treatment; AD, artificially dried; ND, naturally dried; ADM, artificially dried with medium; NDM, naturally dried with medium.

**
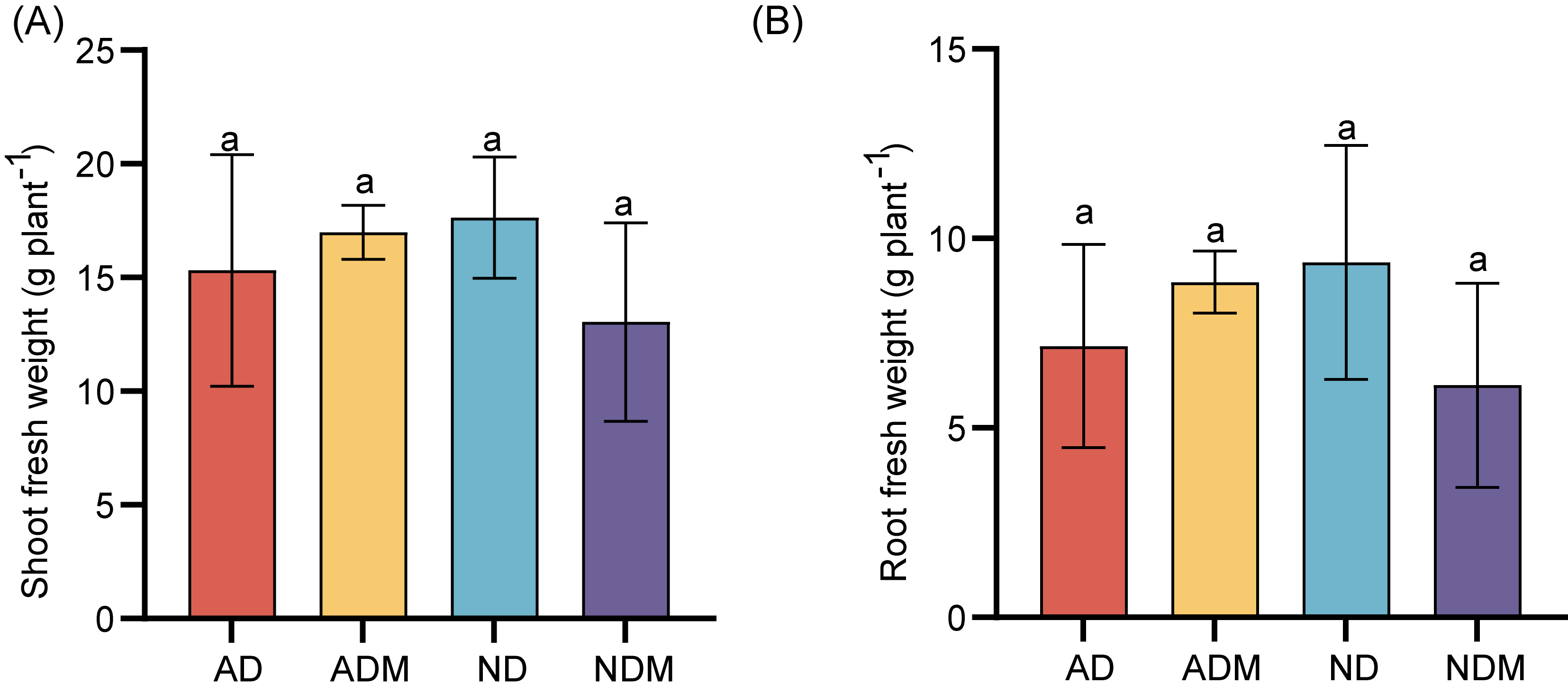
**

**Figure S2** **Fresh weight of maize in different treatments.** (A) Fresh weight of maize shoot. (B) Fresh weight of maize root. AD, artificially dried; ND, naturally dried; ADM, artificially dried with medium; NDM, naturally dried with medium.

**
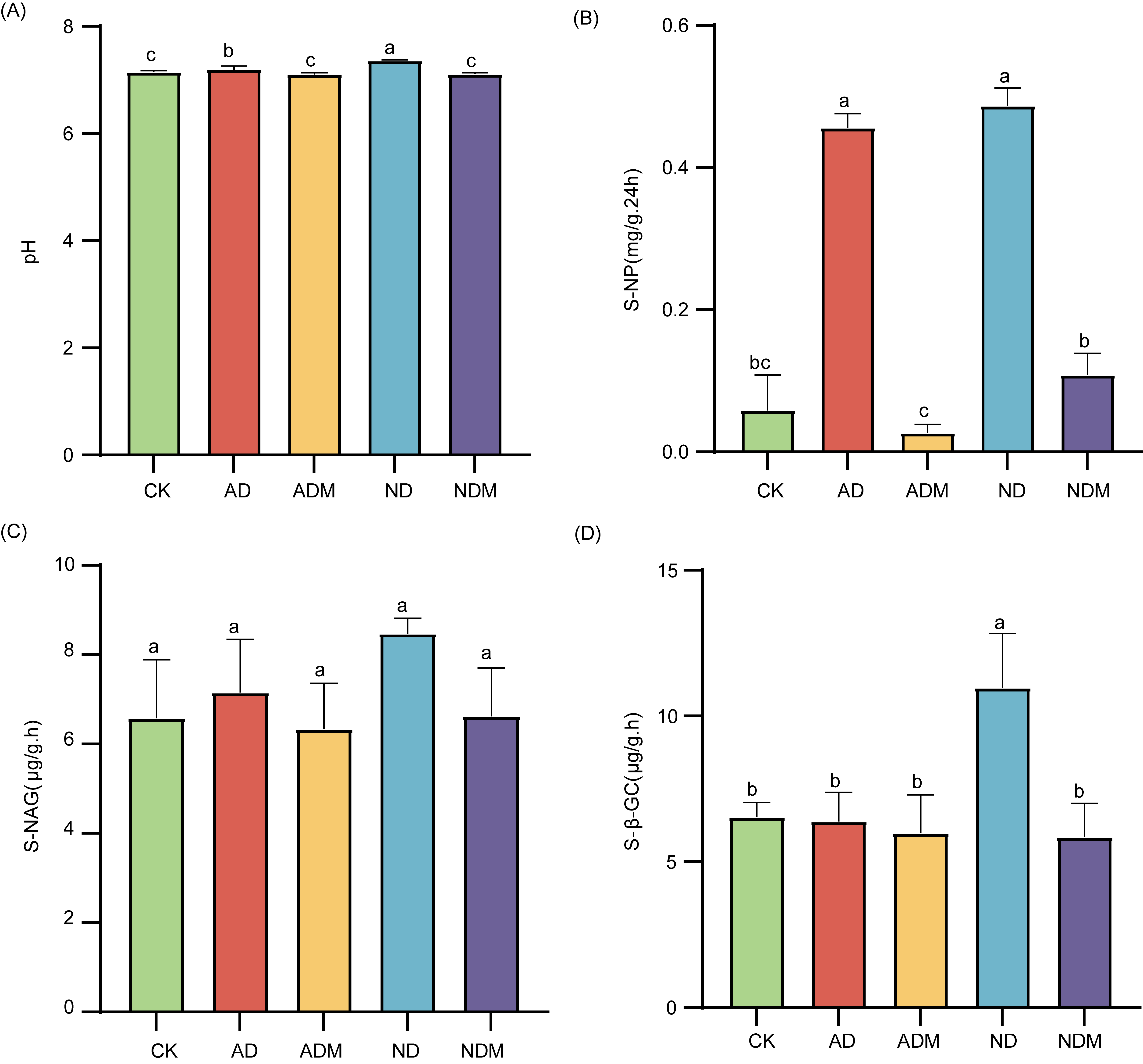
**

**Figure S3 Soil chemical properties in different treatments.** (A) pH of the rhizosphere soil in different treatments. (B) Soil neutral phosphatase (S-NP) of the rhizosphere soil in different treatments. (C) Soil n-acetyl-D-glucosaminidase (S-NAG) of the rhizosphere soil in different treatments. (D) Soil β-glucosyl enzyme (S-β-GC) of the rhizosphere soil in different treatments. Bar charts with various lowercase letters indicate significant levels between different treatments based on the LSD test (*p* < 0.05).


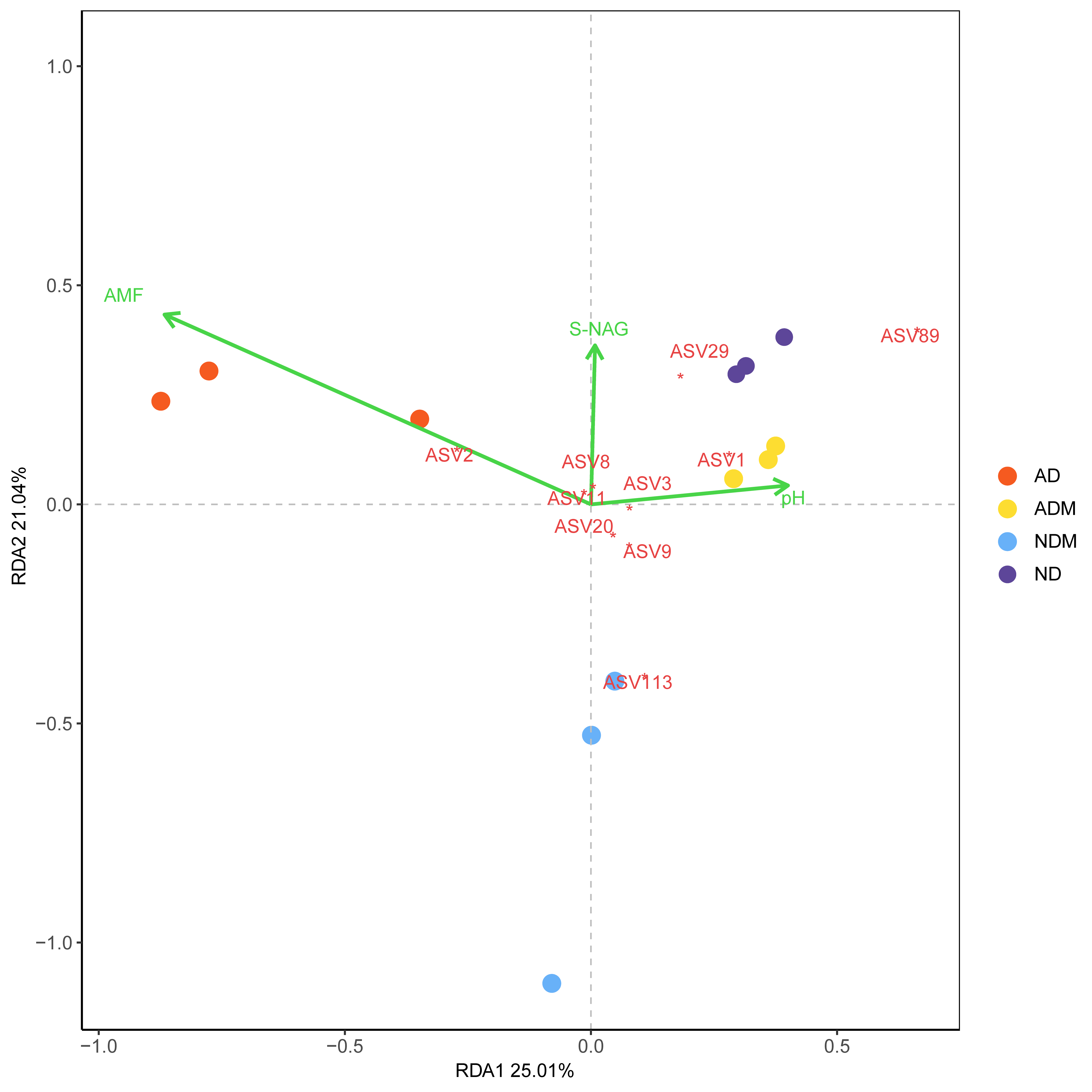


**Figure S4 Redundancy analysis (RDA) illuminating soil chemical properties and bacteria community composition at the phylum level.**


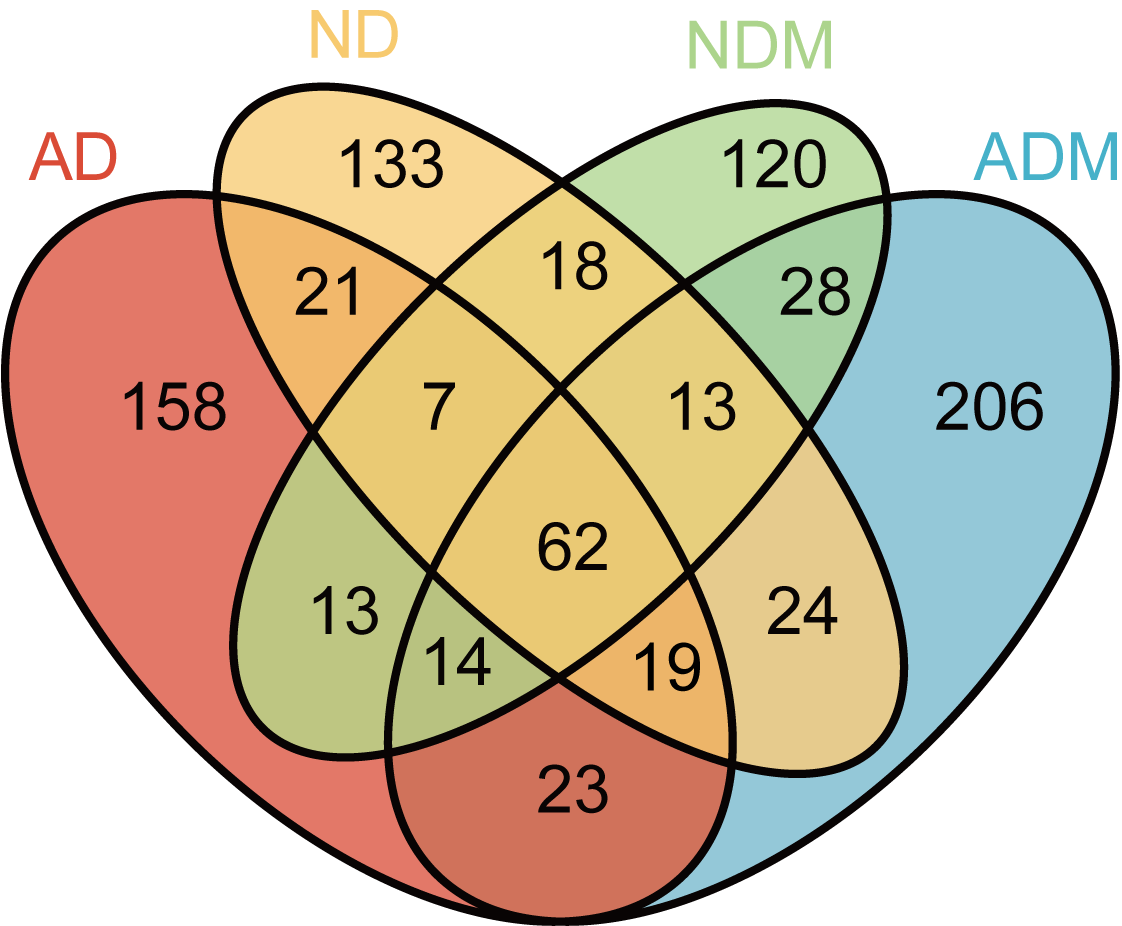


**Figure S5 Venn diagram of endophytic bacterial ASV in each treatment.**

**
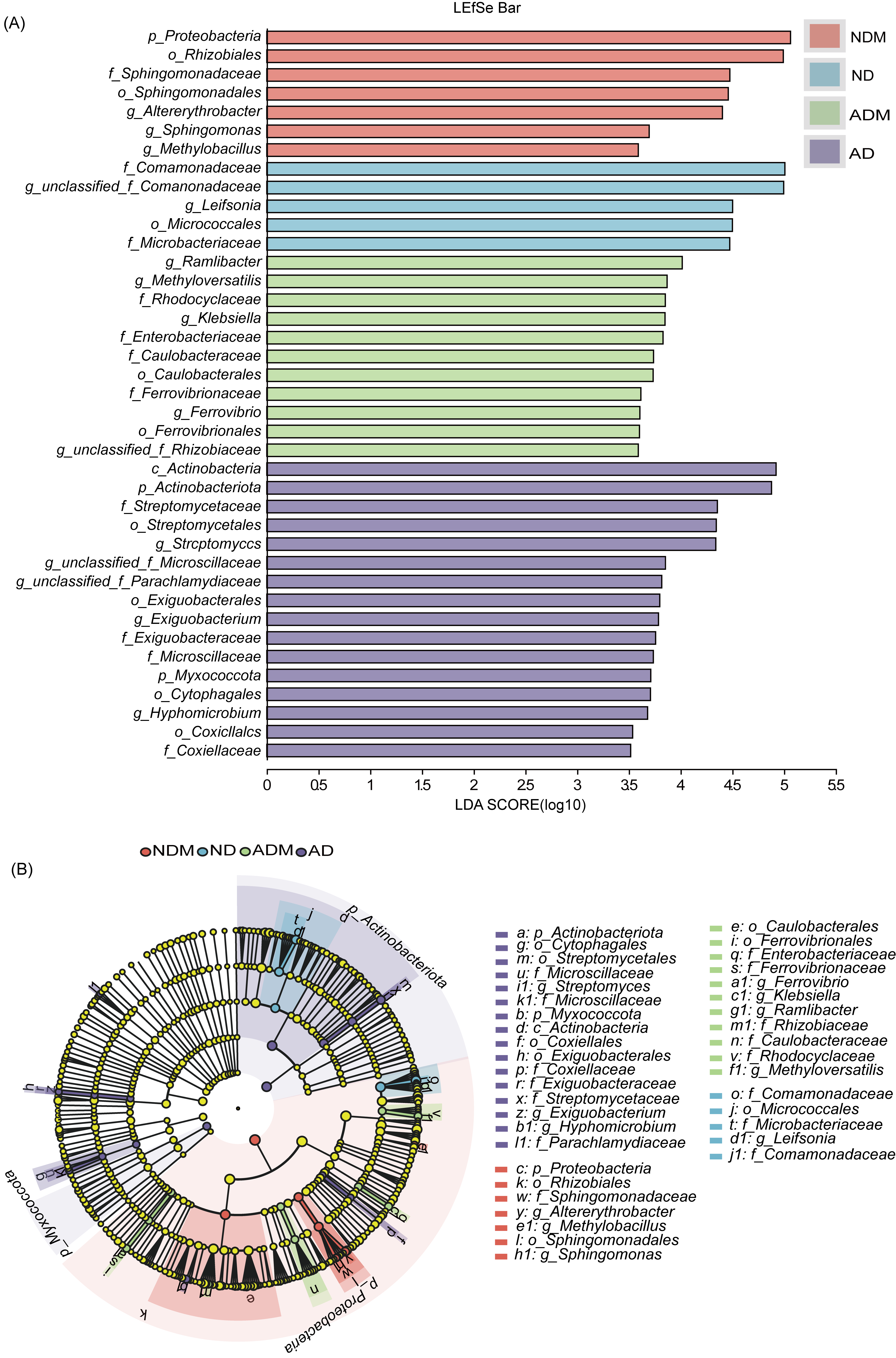
**

**Figure S6** **Linear discriminant analysis effect size (LEfSe) analysis revealed significant differences endophytic bacteria in different groups.** (A) LDA effect size taxonomic distribution revealed significant differences in the abundance of bacteria taxa in different groups. The threshold of LDA score was 3.5. (B) Different colored regions represent different treatments (red for NDM, blue for ND, green for ADM, and purple for AD), and the diameter of the circle is proportional to the relative abundance of the taxa, and the inner to outer circles indicate phylogenetic taxonomic units from phylum to genus.


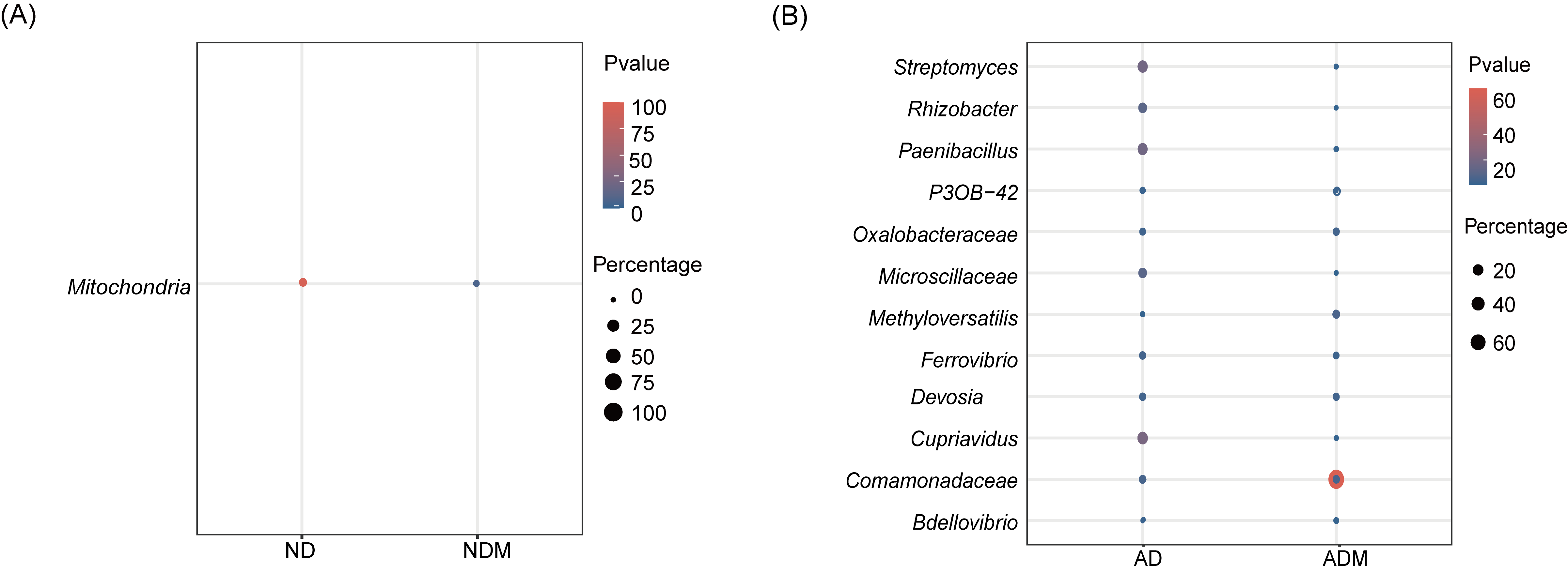


**Figure S7 The relative abundance of bacterial genera with differences is shown in the bubble diagram.** (A) Relative abundance of bacterial genera differing in ND and NDM treatments. (B) Relative abundance of bacterial genera differing in AD and ADM treatments. Larger circles indicate higher abundance.


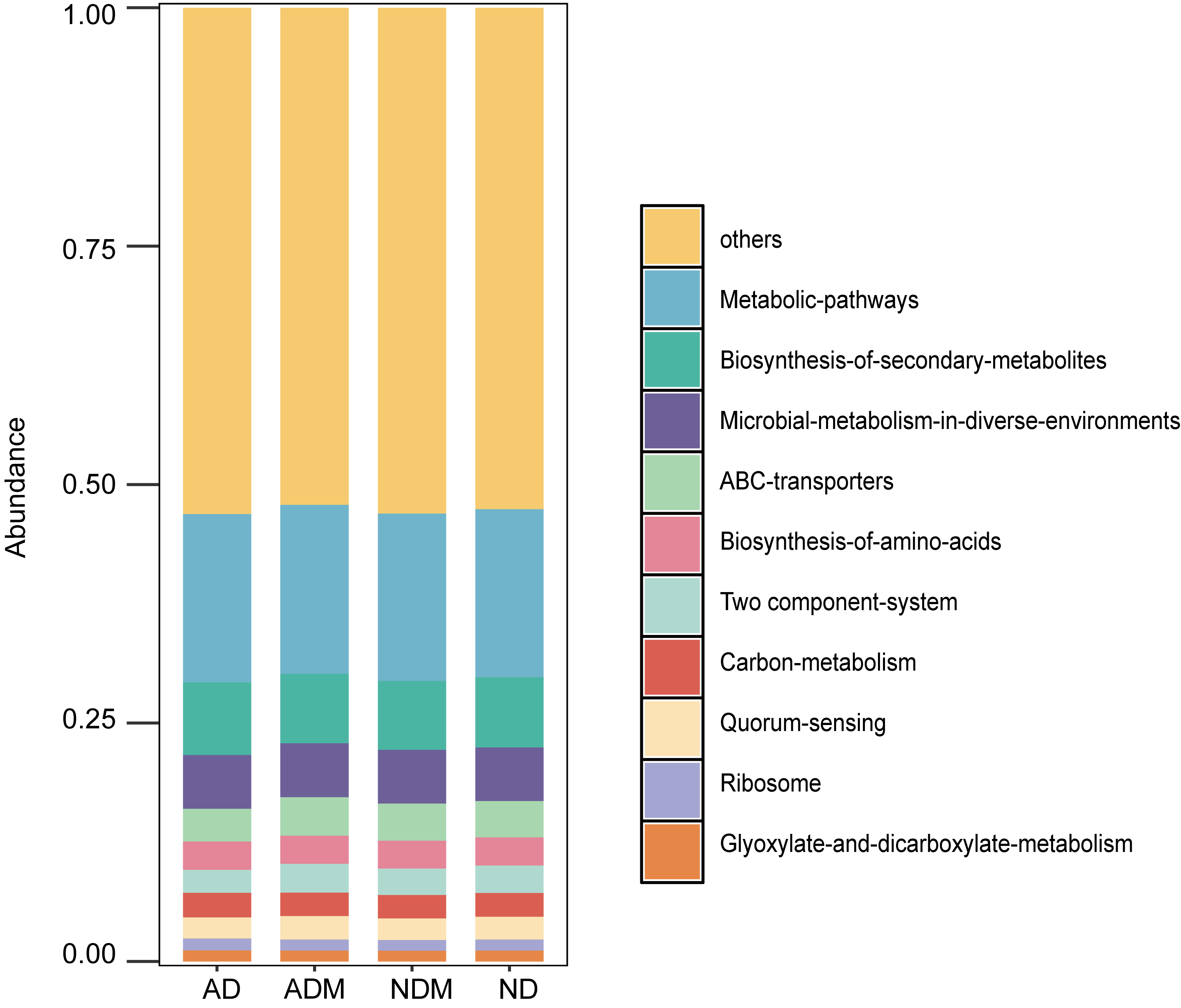


**Figure S8 Functional composition of endophytic bacteria in roots of maize in different treatments.**


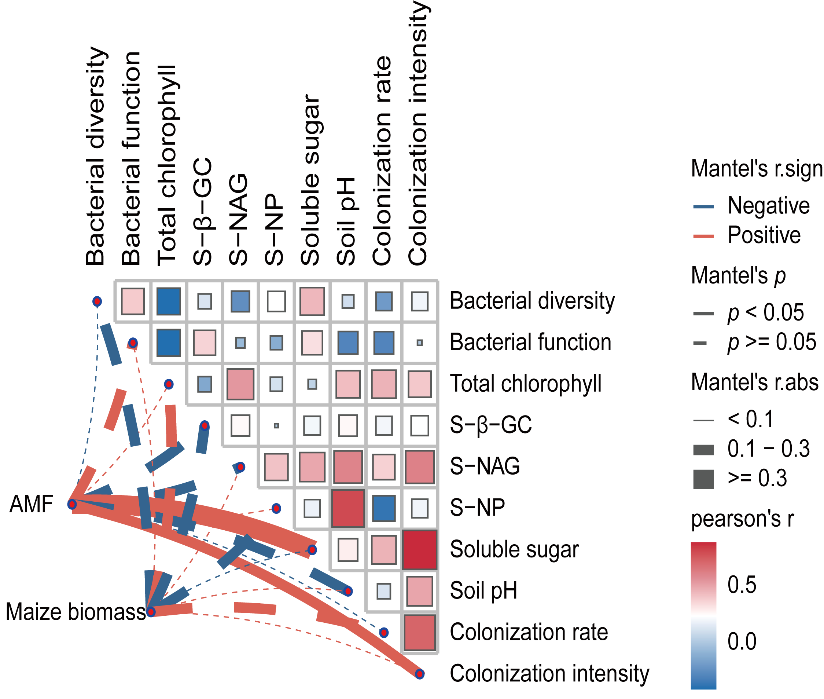


**Figure S9 Mantel test revealed the correlation between bacterial diversity and function, soil physical and chemical properties, maize physiological indexes, and maize biomass and AMF.** The width of the line corresponds to t-Mantel's R statistic of distance correlation, and the color of the line indicates statistical significance. Pearson’s correlations between different environmental factors are indicated by shades of color.
